# Combined Analysis of RRBS DNA Methylome and Transcriptome Reveal Novel Candidate Genes Related to Porcine *Clostridium perfringens* Type C-Induced Diarrhea

**DOI:** 10.3389/fgene.2022.803477

**Published:** 2022-03-25

**Authors:** Xiaoyu Huang, Qiaoli Yang, Zunqiang Yan, Pengfei Wang, Hairen Shi, Jie Li, Xuefeng Shang, Shuangbao Gun

**Affiliations:** ^1^ College of Animal Science and Technology, Gansu Agricultural University, Lanzhou, China; ^2^ Tibet Academy of Agricultural and Animal Husbandry Science, Lasa, China; ^3^ Gansu Research Center for Swine Production Engineering and Technology, Lanzhou, China

**Keywords:** DNA methylome, transcriptome, *Clostridium perfringens* type C, diarrhea, pig, resistance, susceptibility

## Abstract

*Clostridium perfringens* type C (Cp) is one of the principal microorganisms responsible for bacterial diarrhea in neonatal and pre-weaning piglets. To better understand the molecular effects of Cp infection, we performed a genome-wide comparison of the changes in DNA methylation and gene expression in Cp infected resistant and susceptible piglets. We characterized the pattern of changes in methylation and found 6485, 5968, and 6472 differentially methylated regions (DMRs) of piglets infected with Cp in IR vs. IC, IS vs. IC, and IS vs. IR groups, respectively. These methylation changes for genes mainly involved in immune and inflammatory responses, cell adhesion, and activation of transcription factors. Gene ontology and KEGG pathway analyses showed that the differentially methylated genes (DMGs) were associated with negative regulation of transcription, apoptotic processes, protein binding, and kinase activity. In addition, they were enriched in immunity-related pathways, such as MAPK signaling pathway, Toll-like receptor signaling pathway, and NF-kappa B signaling pathway. Integrative analysis identified 168, 198, and 7 mRNAs showing inverse correlations between methylation and expression with Cp infection. Altered DNA methylation and expression of various genes suggested their roles and potential functional interactions upon Cp infection, 14 immune-associated mRNAs with differential methylation and transcriptional repression were identified in IS vs. IR, commonly revealing that the differentially expressed genes (DEGs) *LBP*, *TBX21*, and *LCN2* were likely involved in the piglets against Cp infection. The present results provide further insight into the DNA methylation epigenetic alterations of *C. perfringens* type C infected piglet ileum tissues, and may advance the identification of biomarkers and drug targets for predicting susceptibility to and controlling *C. perfringens* type C-induced piglet diarrhea.

## Introduction

*Clostridium perfringens* (*C. perfringens*) type C (Cp) frequently causes the severe, acute, and lethal necrotic enteritis (NE) in humans and livestock (Rood and Cole 1991; Lyras and Rood 2014), such as calves, sheep, goats, and pigs (Songer and Uzal 2005), especially in newborn piglets (Petit et al., 1999; Rood et al., 2018). Newborn piglets from the birthday until 3 weeks of age are highly susceptible to the clostridia because of their incompletely developed intestinal immune system, leading to mortality rates up to 100%. The Cp infection spreads rapidly and becomes an important problem worldwide (Posthaus et al., 2020).

*C. perfringens* type C beta (CPB) toxin is the essential virulence factor (Sayeed et al., 2008; Vidal et al., 2008; Uzal et al., 2009). Usually, the colonization and rapid proliferation of Cp intruded into the piglet’s incompletely developed intestine forebode the start of NE disease. Due to trypsin inhibitors in the prevention of degradation of CPB, secretions of CPB toxin leads to initial epithelial damage or irritation, the toxin-induced intestinal damages rapidly cause an increase in permeability of vessels in the lamina propria, even the epithelial barrier further disrupted. The change of luminal environment causes acceleration of toxin production, which is absorbed into the systemic circulation and further causes hemorrhage, tissue necrosis, and even enterotoxemia (Posthaus et al., 2020). The Cp diseases can occur in acute and subacute-to-chronic forms (Hogh 1967; Chean et al., 2014). Piglets with acute disease usually appear with several symptoms such as depression, hemorrhagic diarrhea, and death mainly within the first 3 d after birth (Songer and Uzal 2005). Piglets with more protracted subacute-to-chronic clinical features almost have non-hemorrhagic diarrhea, and appear with hypo-immunity, growth reduction, and emaciation (Songer and Uzal 2005). Stoy et al. found that *C. perfringens* type A infection led to the increased expressions of inflammatory related genes (*CCL5*, *NFKBIA*, *IL8*, *IL1RN*, and *TNFAIP3*) of IPEC-J2 cells, and total count and densities of bacteria were markedly high in pigs NE, showing the significant positive correlation with disease severity (Stoy et al., 2015).

DNA methylation is one of the central epigenetic modifications; in mammalian genomes, it mainly occurs on cytosines at position C5 in CpG dinucleotides (Wang et al., 2019). DNA methylation always participates in numerous immunity and physiology processes, such as genomic imprinting, transcriptional regulation, growth, and developmental, immune, and inflammation regulation (Schübeler 2015). The methylation state normally dynamically changes and serves to regulate expressions of the responsive genes during host responses to environmental stimuli of pathogen infection, drug treatment, pollutants, and immune and inflammatory diseases (Kiga et al., 2014; Jiang et al., 2018; Swathy et al., 2018; Chen et al., 2019). Generally, the DNA occurrence of promoter methylation is often accompanied by transcription inhibition (Lee and Bartolomei 2013). Hypomethylation can promote the increase of transcriptional activity (Lluis and Cosma 2013), abnormal methylation can cause many diseases. Research has reported that bacterial endotoxins have profound impacts on gene expression in intestinal epithelial cells through DNA methylation modifications. The expressions of *FUT1* (Dai et al., 2017) and *FUT2* (Wu et al., 2018) were epigenetically modulated by DNA methylation of their promoters, regulating ETEC F18 resistance in weanling piglets. Other studies also addressed the impact of infection and LPS on the DNA methylation status of immune cells. In human macrophages, LPS-induced specific methylation changes lead to inactivation of pro-inflammatory pathways (Novakovic et al., 2016). However, systematic investigations on the global DNA methylation changes induced by *C. perfringens* type C infection and the methylation pattern of responsive genes are still scant.

This study aimed to explore the genomic regions and distribution of DNA methylation in piglet ileum tissues exposed to Cp infection and screened the potential DNA methylation targets for piglets against Cp infection, combined with RNAseq data of our previous study (Huang X. et al., 2019). This study comprehensively analyzed the effects of RRBS DNA methylome level and transcriptome level, and provided new insights in Cp-induced piglet diarrhea disease, which may contribute to the identification of biomarkers for diarrhea resistance against Cp infection.

## Materials and Methods

### Animal Experiment

Bacterial culture, feeding, and management of piglets were in accordance with the description of Huang et al. (Huang X. et al., 2019; Huang X. Y. et al., 2019); the details were as follows: *Cp* strain (CVCC 2032; China Veterinary Culture Collection Center) was anaerobic shaking cultured 16 h at 37°C in the bouillon culture-medium (HopeBio, Qingdao, China), and an expected concentration of 1 × 10^9^ CFU/ml Cp medium was used to inoculate piglets orally.

The 30 7-day-old experimental piglets (Landrace male × Yorkshire female) tested seronegative for *Escherichia coli* (*E. coli*), *Salmonella,* and *C*. *perfringens* by commercial enzyme-linked immunosorbent assay (ELISA) kits (Jiancheng Bioengineering Institute, Nanjing, China) from Dingxi City, Gansu Province of China. Then 25 piglets were randomly orally challenged with 1 ml 1 × 10^9^ CFU/ml Cp medium for 5 consecutive days, the 5 remaining piglets were the control group (IC), and all piglets were housed separately and isolated in climate-controlled and fully air-conditioned, receiving water and diets *ad libitum* (Huang X. et al., 2019; Huang X. Y. et al., 2019).

During Cp infection, summing and ranking total scores of each piglet, according to fecal consistency from 0 to 3 grade (Kelly et al., 1990), meanwhile combining with the clinical signs, the top 5 piglets with the highest and lowest fecal scores were designated as susceptibility (IS) and resistance (IR) groups, respectively. Piglets of IR, IS, and IC groups were humanly slaughtered under barbiturate anesthesia. The ileum tissues were collected to extract DNA (Supplementary Figure S1).

### Nucleic Acid Isolation

According to the manufacturer’s instruction, genomic DNA and total RNA were exacted by using the QIAamp Fast DNA Tissue Kit (Qiagen, Dusseldorf, Germany) and TRIzol reagent (Invitrogen, United States). Qualities and integrities of RNA extracts were assessed using the NanoPhotometer® spectrophotometer (Thermo Scientific, United States) and by 1% agarose gel electrophoresis with RNA Nano6000 Assay Kit of the Bionalyzer 2100 system (Agilent Technologies, United States), which were used for library preparation and subsequent analysis.

### Library Preparation and RRBS Sequencing

The 200 to 1,000 bp in length fragmented DNA samples by MspI enzyme (NEB, United States) were then subjected to bisulfite conversion for converting any unmethylated cytosine to Uracil by EZ DNA Methylation-Gold™ Kit (Zymo Research, United States). Further, the Accel-NGS Methyl-Seq DNA Library Kit (Swift, MI) was utilized for attaching adapters to single-stranded DNA fragments. Bead-based SPRI clean-ups were used to remove both oligonucleotides and small fragments, as well as changing enzymatic buffer composition. Finally, we performed the pair-end 2 × 150 bp sequencing on an Illumina Hiseq 4000 platform housed in the LC Sciences (Hangzhou, China).

### Data Normalization

Sequencing reads that contained adapter contamination, low quality bases, and undetermined bases were removed using Cutadapt and perl scripts. Quality control was verified using FastQC v0.11.4 (http://www.bioinformatics.babraham.ac.uk/projects/fastqc/) (Cock et al., 2010), then reads were mapped and aligned to *Sus scrofa* 11.1 reference genome using Bismark v0.22.1 (Krueger and Andrews 2011). Further, for each cytosine site (or guanine corresponding to a cytosine on the opposite strand) in the reference genome sequence, the DNA methylation levels were determined by the ratio of numbers of reads supporting C (methylated) to that of total reads (methylated and unmethylated) using per scripts in house and MethPipe (Song et al., 2013). Analysis of differentially methylated regions (DMRs) was calculated by R package-MethylKit (Akalin et al., 2012) with default parameters (1000 bp slide windows, 500 bp overlap, *p* value < 0.05).

### The Conjunctive Analysis of RRBS and RNA-Seq

Gene promoter DNA methylation usually inhibits gene expression. To explore the effect of DNA methylation on gene expression during piglets suffering from Cp infection, we also had conjunctively analyzed the negative relationship between promoter DMGs and differentially expressed genes (DEGs). Overlapping analysis was performed for DMGs and DEGs, screening negative correlation between gene expression levels and methylation levels by Pearson correlation analysis. The methylation level of DMGs (|ΔMethylation| > 5%) and expression level of DEGs (|Δexpression log_2_FC| > 1) both with the *p* < 0.05 were selected to perform the conjoint analysis.

### Bioinformatics Analyses

Gene ontology (GO) analysis of DMG-associated genes was performed using DAVID functional annotation tool (Huang et al., 2009). All annotated genes in *Sus scrofa* genome were used as background for GO analysis. Pathway enrichment analysis was performed using the Kyoto Encyclopedia of Genes and Genomes (KEGG) database available within the DAVID platform, and with WikiPathways database (https://www.wikipathways.org/) (Martens et al., 2021).

### Data Statistics and Analysis

The *t*-test analysis and one-way ANOVA analysis were used to analyze the significance of diarrhea scores and fecal bacteria shedding of piglets in the IR, IS, and IC groups through SPSS 21.0 software. All values were expressed by mean ± standard error (M±SE), the *p* < 0.05 means the significant difference, *p* < 0.01 means the obviously significant difference.

## Results

### Diarrhea Scores and Fecal Bacteria Shedding of Piglets in the IR, IS, and IC Groups

The average diarrhea score and total diarrhea score of piglets among the IR, IS, and IC groups were statistically analyzed, and the results are shown in Table 1. The average diarrhea scores of piglets in the IR and IS groups were 2.01 ± 0.26 and 2.68 ± 0.04, respectively, which were significantly higher than that in the IC group (0.61 ± 0.02) (*p* < 0.01). The total diarrhea scores of piglets in the IR and IS groups were 37.60 ± 0.87 and 67.6 ± 1.21, respectively, which were significantly higher than that in the IC group (11.40 ± 0.51, *p* < 0.01). These results suggested that the average diarrhea scores and total diarrhea scores of the IR and IS groups were significantly increased after Cp infection (*p* < 0.01).

**TABLE 1:** Analyses of the diarrhea scores of piglets among the IR, IS, and IC groups.

Group	IC	IR	IS
Average diarrhea scores	0.61 ± 0.02^C^	2.01 ± 0.26^B^	2.68 ± 0.04^A^
Total diarrhea scores	11.40 ± 0.51^C^	37.60 ± 0.87^B^	67.6 ± 1.21^A^

**FIGURE 1:**
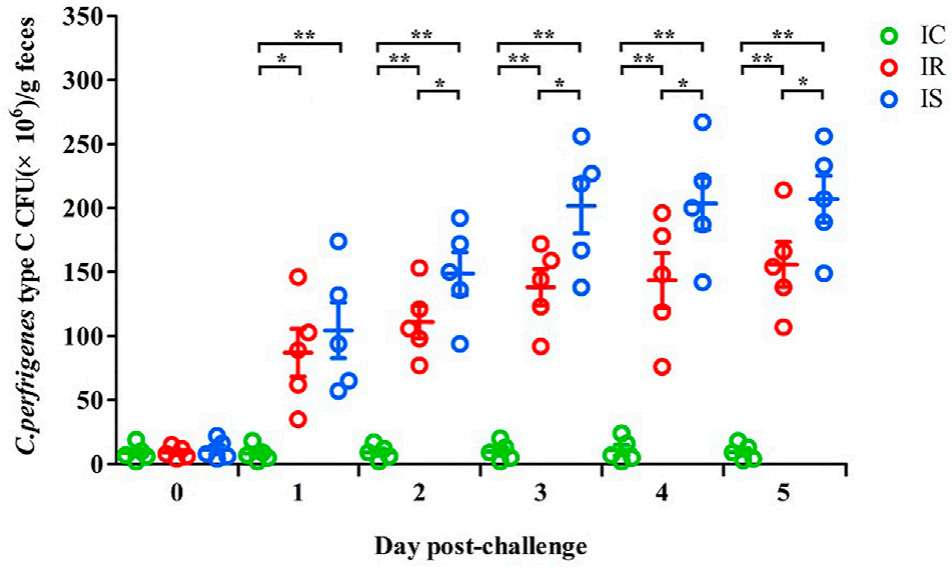
The fecal shedding levels of piglets in the IR, IS, and IC groups after Cp infection. Note: Fecal CFUs were determined by plate count method. The horizontal line represents the mean. Green circles represent the IC group, red circles represent the IR group, and blue circles represent the IS group. An asterisk denotes a significant difference (*p < 0.05, **p < 0.01).

The numbers of piglet feces Cp in the IR, IS, and IC groups were also counted, which are shown in Figure 1. As increasing times of Cp infection, the numbers of Cp in feces of piglets in the IR and IS groups were significantly increased, meanwhile, the numbers of feces Cp of piglets in the IS group were also significantly higher than those in the IR group (*p* < 0.05), which were both significantly higher than those in the IC group (*p* < 0.01). The results showed that Cp infection led to increasing numbers of feces bacteria of piglets, the tolerant piglets in the IR group had stronger resistance to bacterial infection, showing numbers of feces bacteria shedding obviously less than those in the IS group.

### DNA Methylation Profiles of RRBS

Genome DNA methylation profiles of quintuplicate samples of IR, IS, and IC groups were analyzed. Overall, RRBS yielded an amount of 39–49 million reads per sample. After quality filtering, ranging from 59.6–65.63%, average 61.23% of reads were successfully aligned to the *Sus scrofa* 11 reference genomes. In total, we had identified 1.7–2.2 million CpG sites per sample, of which, the average 1.96 million were covered in all samples, representing 7.9% of total numbers of CpGs in the *Sus scrofa* genome. Raw sequencing data and mapping statistics are summarized in Supplementary Table S1.

To detect the DNA methylome changes induced by Cp infection, we compared Cp infected and uninfected piglets to identify methylated enrichment peaks (MEPs) in genomic DNA (Supplementary Table S2). Statistically, compared to the IC group, there were 157,833 MEPs identified in the IR group (*p* < 0.05), of which 110,874 were hypermethylated and 46,959 were hypomethylated (Supplementary Table S3), and 150,128 MEPs were identified in the IS group (*p* < 0.05), of which 79,818 were hypermethylated and 70,310 were hypomethylated (Supplementary Table S4). Mentionable, compared to the IR group, 160,738 MEPs were identified in the IS group (*p* < 0.05), of which 52,995 were hypermethylated and 107,743 were hypomethylated (Supplementary Table S5).

**FIGURE 2:**
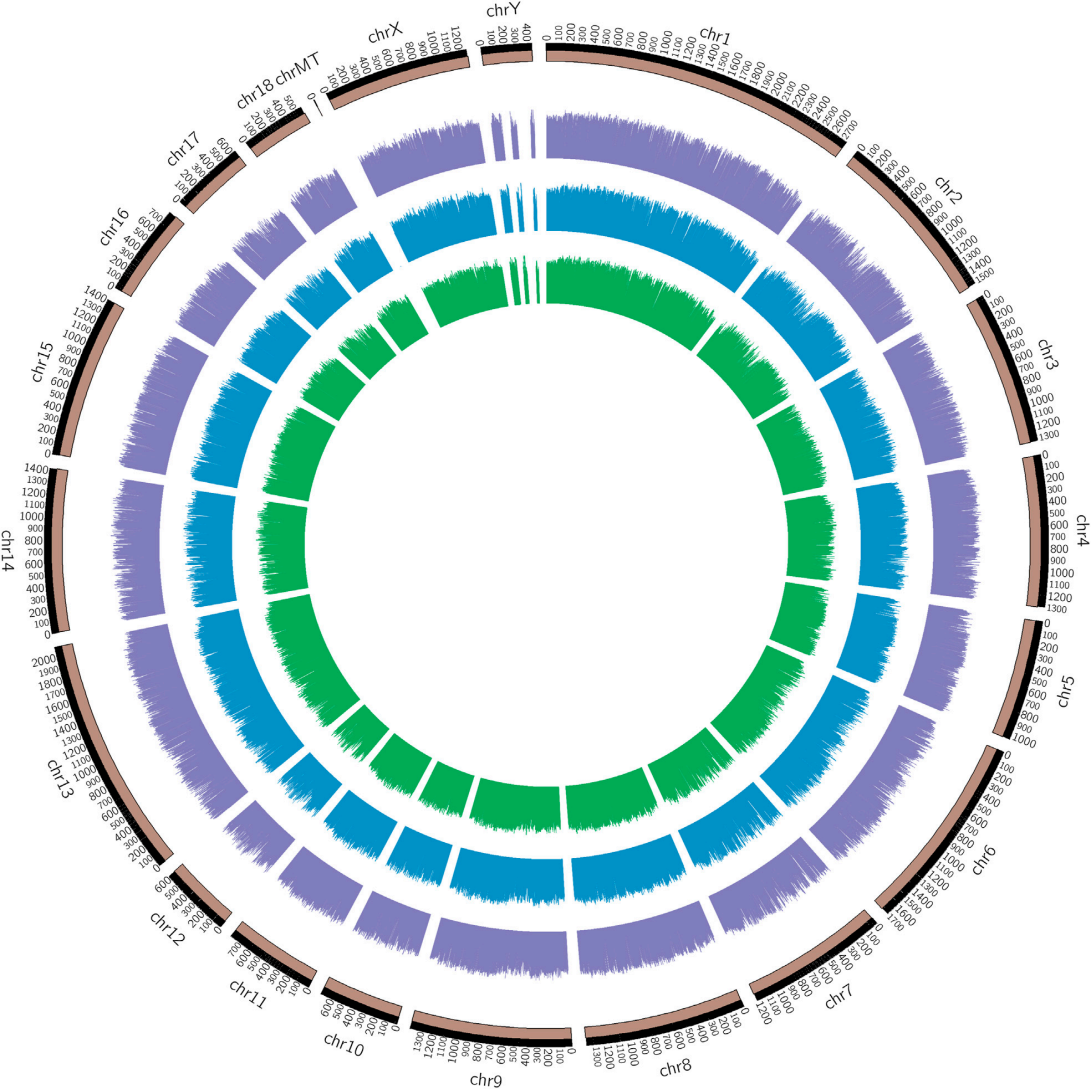
Genomic distribution of DMRs among Cp infected and uninfected piglets. Note: From the inner circle to the outer circle represents the IR vs. IC, IS vs. IC, and IS vs. IR groups.

The chromosomal distribution of the methylated peaks was determined to assess whether methylation was associated with specific chromosomal features. As shown in the methylation map (Figure 2), the distribution of identified methylations almost covered all chromosomal, the methylation density in these regions was distinct among the chromosomes, chromosome MT (mitochondria) in particular, contained a relatively large unmethylated region among samples, which was related to the different degrees and correlation between methylation profiles of the infected IR and IS groups and the uninfected IC group.

Based on the CpG ratio, GC content, and length of the CpG-rich region, we divided gene promoters into three types: High-density CpG promoter (HCP), low-density CpG promoter (LCP), and intermediate density CpG promoter (ICP) (Yu et al., 2013). The information of MEPs in three comparative groups were shown and revealed the relatively uneven distribution across the genome. The majority of MEPs were in the intergenic regions, following in the exon and intron, lowest in the promoter (Table 2).

**TABLE 2:** Analysis and distribution of the significant methylated genes of the IR, IS, and IC groups.

	IR vs. IC		IS vs. IC		IS vs. IR	
	Hyper-methyl	Hypo-methyl	Hyper-methyl	Hypo-methyl	Hyper-methyl	Hypo-methyl
Promoter	15,934	5,396	10,268	9,066	6,402	15,995
Exon	23,456	8,894	16,261	14,012	10,289	22,678
Intergenic	54,161	25,762	41,121	36,791	28,458	52,589
Intron	17,272	6,907	12,168	10,441	7,846	16,481

Then distribution of the MEPs in three types of promoter were analyzed. We found that the numbers of MEPs were obviously increased in the IR and IS groups after *C. perfringens* type C infection, which included MEP in LCP types, followed by in HCPs and ICPs. It is worth attention that in promoter CGIs, HCPs had more MEPs in the IS vs. IR group than ICPs or LCPs (Table 2).

### Methylation Status of Genome CpG Islands

**FIGURE 3:**
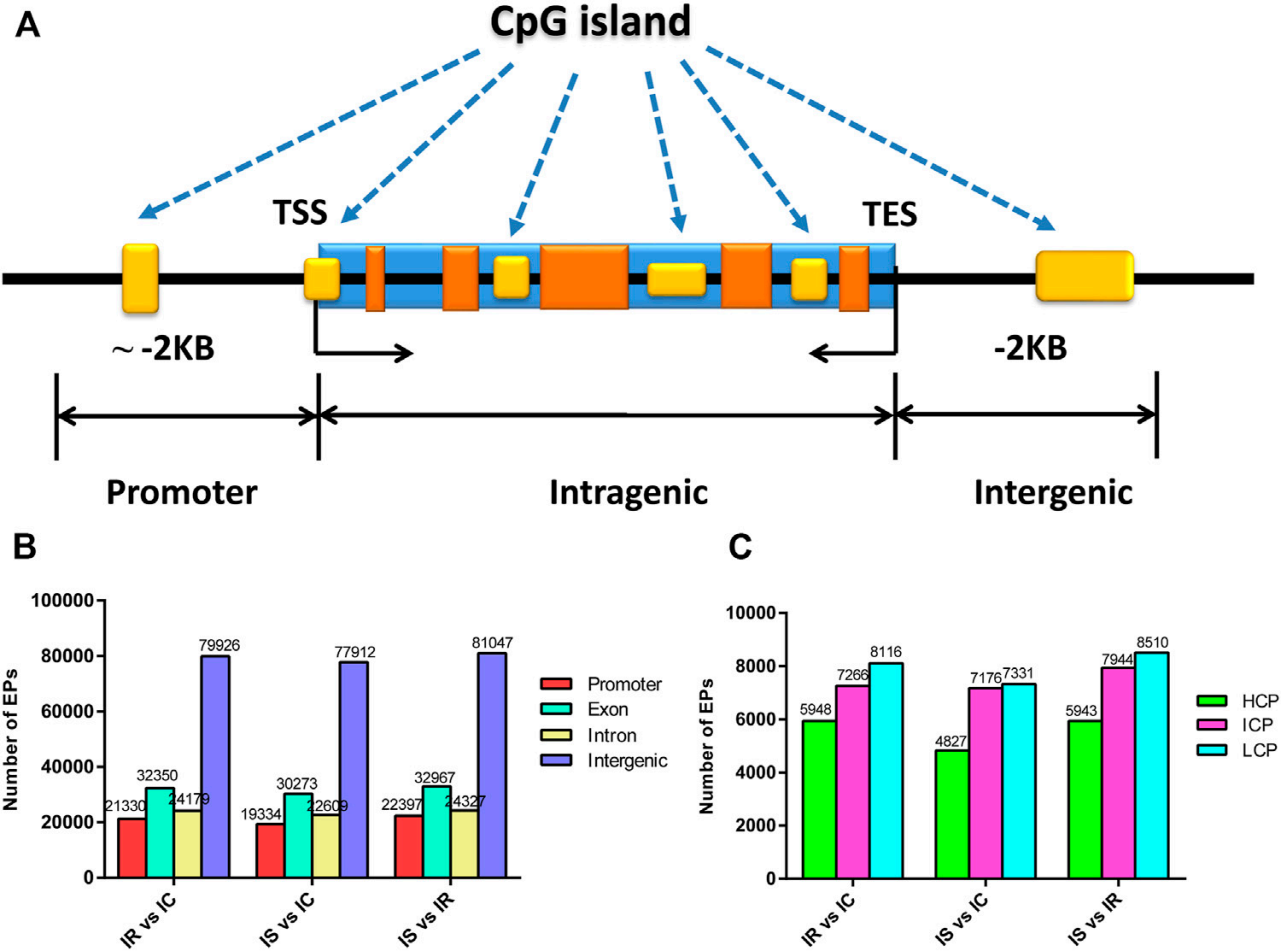
Distribution of DNA methylation enrichment peaks in piglet ileum infected with *C. perfringens* type C infection. **(A)** Generic diagram showing CpG islands (CGIs) relative to gene transcript regions. **(B)** Numbers of MEPs in the CGI region of IR, IS, and IC groups. **(C)** Numbers of MEPs in the promoter CGIs. Note: HCP represents high CpG density promoter; ICP represents intermediate CpG density promoter; LCP represents low CpG density promoter.

CpG islands (CGIs) obtained particular attention and interest for their role in controlling gene expression through epigenetic modification. We grouped the CGIs into four classes according to their distance to the RefSeq genes: promoter CGIs [from about −2 kb to +0.5 kb around the transcription start site (TSS)], exon and intron CGIs [from +0.5 kb around the TSS to the transcription terminal site (TTS)], and intergenic CGIs (about 2 kb to those that do not fall into either the promoter or the intragenic group) (Figure 3A).

We also analyzed the CpG methylation status of different gene segments in the piglet ileum after Cp infection. The numbers of MEPs in the four classes of CGIs and CGIs shore among IR, IS, and IC groups are shown in Figures 3B,C. Most MEPs were distributed in the intergenic CGIs among IR, IS, and IC comparative groups. It was worth mentioning that more CGI methylations happened in the IR vs. IC group (Supplementary Table S6) and the IS vs. IC group (Supplementary Table S7) than in the IS vs. IR group (Supplementary Table S8) (*p* < 0.05). Importantly, for the promoter CGIs, intergenic CGIs and 3′ transcript CGI, the numbers of hyper MEPs were also higher than hypo MEPs in the IR vs. IC group and the IS vs. IC group, which was opposite to those in the IS vs. IR group (Figure 3B; Table 3).

**TABLE 3:** The distributions of the significant CGIs methylations of the IR, IS, and IC groups.

	IR vs. IC		IS vs. IC		IS vs. IR	
	Hyper-methyl	Hypo-methyl	Hyper-methyl	Hypo-methyl	Hyper-methyl	Hypo-methyl
Promoter CGI	2,579	759	1,520	1,450	885	2,574
Intragenic CGI	14,535	5,312	9,854	8,597	6,191	13,882
3′ transcript CGI	1,489	435	913	749	505	1,418
Intergenic CGI	28,660	11,363	19,980	17,818	12,822	27,933

Note: CGIs represents CpG islands; CGIs shore represents CpG island shore.

### Identification of Differentially Methylated Genes

**FIGURE 4:**
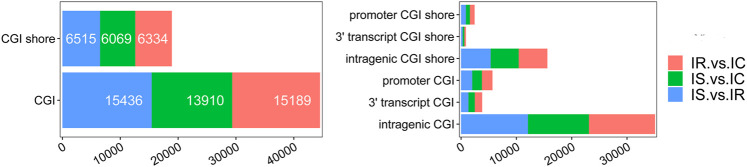
The details of CGIs and CGI shores distributed in the IR vs. IC, IS vs. IC, and IS vs. IR groups.

**FIGURE 5:**
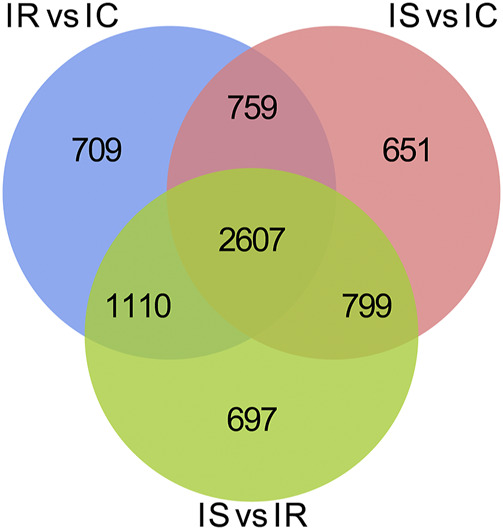
Venn distribution of the differentially methylated mRNAs with CGIs in the IR vs. IC, IS vs. IC, and IS vs. IR groups. GO and KEGG signaling pathway analyses.

To explore the DMGs of piglet ileum tissues induced by Cp infection, we subsequently mapped all DMRs to their nearest genomic features and analyzed the DMGs located in promoter and CGI regions. Compared to the IC group, there were 6,485 DMGs having one or more DMRs including 5,186 mRNAs, 14 miRNA, and 989 pseudogenes in the IR group (Supplementary Table S6), 5,968 DMGs including 4,819 mRNAs, 6 miRNA, and 886 pseudogenes in the IS group (Supplementary Table S7), meanwhile, compared to the IR group, 6,472 DMGs including 5,214 mRNAs, 14 miRNA, and 958 pseudogenes in the IS group (Supplementary Table S8). Except for miRNA and pseudogenes, DMGs almost contained an average of 4 DMRs in the gene body and promoter regions, mainly distributed in CGI and CGI shore (Figure 4). In total, 2607 DMGs were detected among the IR vs. IC, IS vs. IC, and IS vs. IR comparative groups (Figure 5).

**FIGURE 6:**
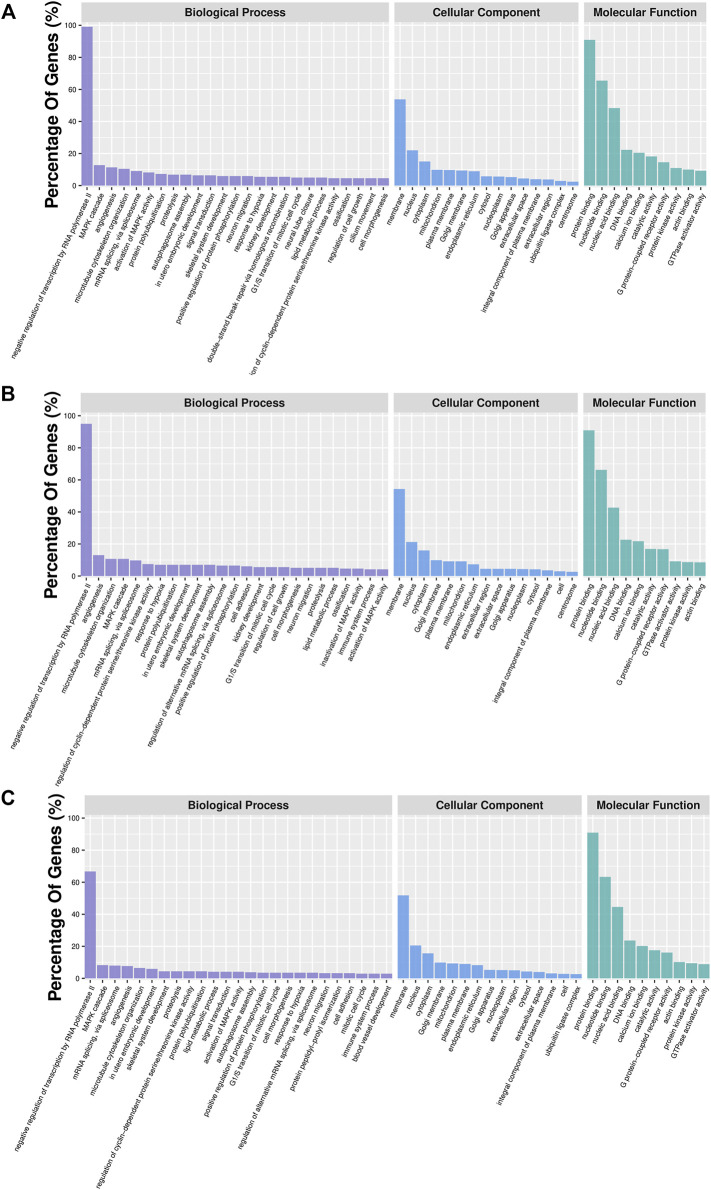
GO analyses of DMGs located in the vicinity of significant differentially methylated regions (DMRs) of the IR vs. IC **(A)**, IS vs. IC **(B)**, and IS vs. IR groups **(C)**. Bar plots display enriched GO terms. The plots show significantly enriched degrees (*p* < 0.05).

In order to further characterize DMGs, gene ontology and KEGG signaling pathway analyses were carried out. GO analysis revealed 46, 35, and 38 GO terms significantly enriched in the IR vs. IC, IS vs. IC, and IS vs. IR groups (Supplementary Table S9), respectively. We found that Cp infection caused the DMGs mainly enriched in the biological and molecular functions, such as signal transduction, ion channel activity, nucleotide binding, protein transferase activity, immune response, cytoskeleton, GTPase activator activity, histone deacetylase complex, inactivation of MAPK activity, and nucleotide immunoglobulin production (Figure 6).

**FIGURE 7:**
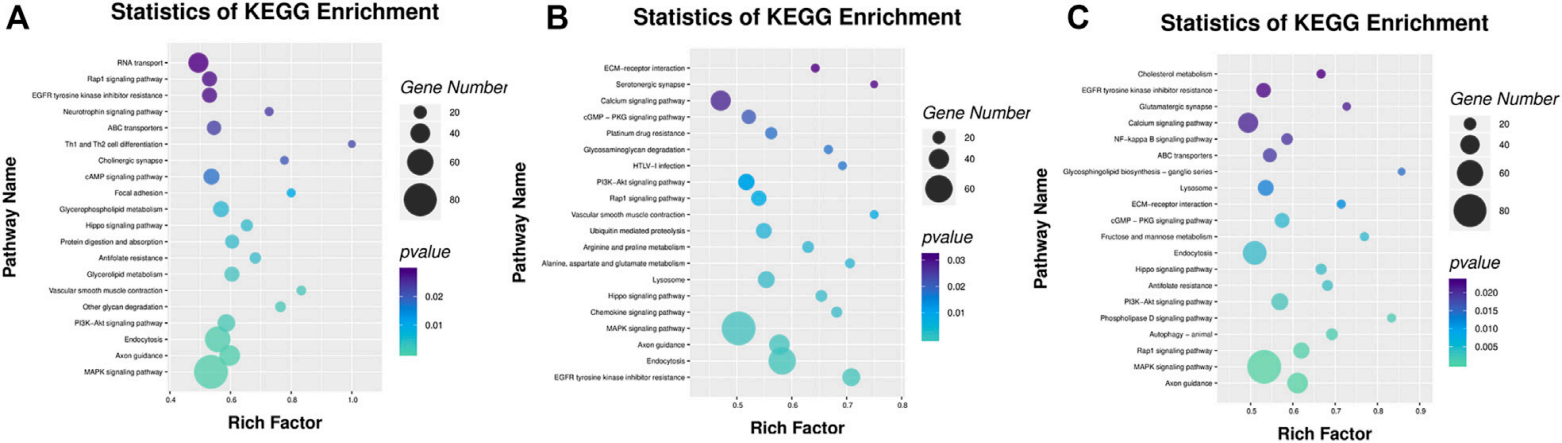
KEGG signal pathway analyses of DMGs located in the vicinity of significant differentially methylated regions (DMRs) of the IR vs. IC **(A)**, IS vs. IC **(B)**, and IS vs. IR groups **(C)**. Bar plots display enriched KEGG pathways. The plots show significantly enriched degrees (*p* < 0.05).

Among the KEGG pathway enrichment, a total of 23, 25, and 29 pathways were significantly enriched in the IR vs. IC, IS vs. IC, and IS vs. IR groups (Supplementary Table S10), respectively. The DMRs were enriched by MAPK signaling pathway, NF-kappa B signaling pathway, tight junction, chemokine signaling pathway, calcium signaling pathway, lysosome, PI3K-Akt signaling pathway, autophagy, Toll-like receptor signaling pathway, ECM-receptor interaction, and several metabolism pathways (Figure 7).

### Correlation Analysis Between DNA Methylation and Gene Expression

DNA methylation of gene promoters is usually involved in inhibiting the expression level of the corresponding genes (Jones 2012). The potential effects of DNA methylation on gene expression were characterized by comparing methylation and RNA expression data (Guo et al., 2019). By analyzing a wide association between transcriptome gene expression and epigenome DNA methylation (in promoter or body), we explored the relationship between methylation changes at the promoter regions and gene expression changes (our previous study) of the IR, IS, and IC groups (Huang X. et al., 2019; Huang X. Y. et al., 2019).

**FIGURE 8:**
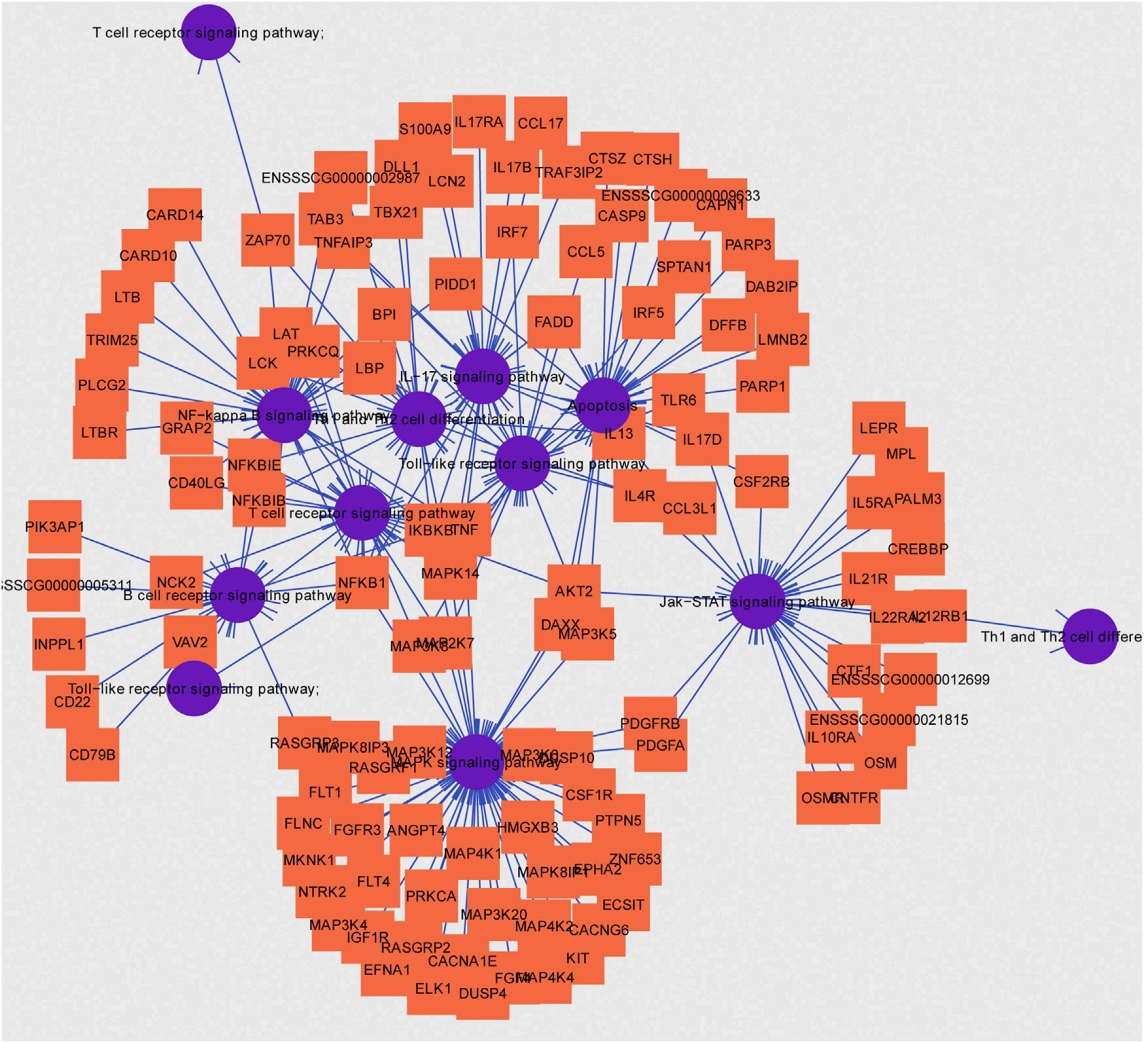
Hub pathways and associated genes in piglets of the IS vs. IR group suffering from *C. perfringens* type C infection.

Usually, there is the significant negative association between mean methylation levels of promoter regions and expressions of mRNAs. According to methylation levels of DMGs (|ΔMethylation| > 5%) and expression levels of DEGs (|Δexpression log_2_FC| > 1) both with the *p* < 0.05, there were 168 mRNAs, 198 mRNAs, and 7 mRNAs screened in the IR vs. IC, IS vs. IC, and IS vs. IR groups, respectively (Supplementary Table S11). Importantly, to further reveal the functions of epigenetics and transcriptomics during piglets’ resistance to Cp infection, we also analyzed the immune-associated mRNAs in the IS vs. IR group and constructed a differential methylation and transcription network (Figure 8). In this network, the screened mRNAs mainly belonged to T cell receptor, Toll like receptor, NF-κB, MAPK, JAK-STAT, IL-17, Th1, and Th2 cell differentiation signaling pathways, such as *NFKB1*, *MAPK14*, *TRIM25*, *TLR6*, *IL21R*, *LBP*, *IRF7*, *TBX21* and so on. Moreover, there were 14 mRNAs, which promoter methylations were obviously inversely related to transcriptional repressions, commonly identified (Table 4), in which *LCN2* with down-expressed and hypomethylated, *AKT2* with up-expressed and hypermethylated, were also identified in the IR vs. IC and IS vs. IC groups.

**TABLE 4:** List of differentially methylated and expressed mRNAs in *C. perfringens* type C infected piglets of the IR vs. IS group.

Gene name	Expression change	Methylation change
*CCL17*	Upregulated	Hypermethylated
*LBP*	Downregulated	Hypomethylated
*ENSSSCG00000002987*	Downregulated	Hypomethylated
*LCN2*	Downregulated	Hypomethylated
*FLNC*	Upregulated	Hypomethylated
*AKT2*	Upregulated	Hypomethylated
*ENSSSCG00000012699*	Upregulated	Hypomethylated
*ZAP70*	Upregulated	Hypomethylated
*NTRK2*	Downregulated	Hypermethylated
*CD40LG*	Upregulated	Hypermethylated
*TBX21*	Upregulated	Hypermethylated
*CASP9*	Upregulated	Hypermethylated
*IL22RA2*	Upregulated	Hypermethylated
*PALM3*	Upregulated	Hypermethylated

## Discussion

In this study, we have revealed the epigenetic alterations in piglet ileum tissues due to infection by Cp from a genome-wide comparative methylome analysis. However, it may only provide limited insights into the biological mechanisms of piglet diarrhea induced by Cp infection. Generally, complementary effects and synergistic interactions between omics in life science can be captured by integrative studies of multiple molecular layers. Building upon the successes in single-omics research, a better understanding of the molecular functions and disease etiologies by multi-omics integrated approaches from different omic levels (e.g., genetics, epigenetics, mRNA transcripts, proteins, and metabolites), as well as their interrelations and combined influences on the disease processes (Sun and Hu 2016). Therefore, we further conducted an integrated analysis of RRBS and RNA sequencing data and identified a subset of genes that was implicated in the piglet response to Cp infection.

The host immune response is crucial for defense against microbial pathogens, it is not different to that found in the complex process of host-pathogen interactions, the genomic expression pattern and program of host reflects responses to pathogens and virulence (Boldrick et al., 2002). Recently, a study reported that epigenetic modulations such as host DNA methylation could be manipulated to influence the host’s gene expressions in response to defensing pathogens infection (Paschos and Allday, 2010). In addition, the changes and differences of DNA methylation presumably largely reflected the abilities of host epigenetic responses involved in the immune system against or triggers by pathogens (Tarakhovsky 2010). In this study, we have revealed the epigenetic alterations in piglet ileum tissues due to infection by Cp from a genome-wide comparative methylome analysis.

Our study found that the methylated peaks DNA methylation almost covered most chromosomal regions and presented obviously distinct methylation densities (Figure 2), indicating that Cp infection could trigger changes of DNA methylation of candidates. These changes may be correlated with piglets responding to Cp infection. Analysis of differential methylation genes revealed that 6635 DMGs were identified in piglets after Cp infection, in which 3,366 DMGs were identified both in the IR and IS groups (Figure 5), suggesting that the common DMGs identified in piglets of the IR and IS groups had an amount of similar methylation patterns during Cp infection, and further altered expressions of immune-related genes. Meanwhile, 697 DMGs especially identified in the IS vs. IR group (Figure 5), illustrating Cp-infected tolerant and sensitive piglets appeared with different methylation patterns induced by the Cp strain. These identified genes involved in the immune system and bacterial infection have been well studied. For example, *JAK1*, *JAK2,* and *JAK3*, found in three comparable groups, were the prototypical members of the JAK family and could play an essential signaling role for cytokines and interferons involved in immunity and antiviral responses (Ferrao et al., 2018). *RASGRF1*, found in IS vs. IC and IS vs. IR comparative groups, mainly participated in the Ras signaling pathway (Manyes et al., 2014). *AIRE* was one of the important regulators of peripheral T cell homeostasis, and played a certain role in control of intestinal tolerance (Kekalainen et al., 2015). Activation of *AKT2* was considered to protect mice from defense against *Salmonella* enterica Typhimurium infection (Kum et al., 2011), even blocking the development of intestinal inflammatory disease (Liu et al., 2019). These differential DMGs may be the crucial resistance candidates of piglet diarrhea.

The candidate mRNAs modified by differential DNA methylation merit greater attention. Further, combining the promoter methylation gene and its mRNA expression level, we had constructed the networks with differential methylations of DMGs and differential expressions of DEGs (Supplementary Table S11), and screened many immune related mRNAs, *S100A9*, *TARBP2*, *TRIM25*, *TBX21*, *LBP*, *TLR6*, involving the piglets resistant to Cp-induced diarrhea disease through defense-related signaling pathways of T cell receptor, Toll-like receptor, NF-κB, MAPK, and so on. Furthermore, these screened mRNAs’ methylation of their promoters was inversely related to transcriptional repression in piglets after Cp infection. *S100A9* gene is considered to play crucial functions in participating innate immunity and mediating the inflammatory response during infection-induced host inflammation (Ometto et al., 2017). Researchers found S100A8/A9 recombinant attenuated bacterial adherence and invasion (Wang et al., 2018), and inhibited growth of multiple species, including *E. coli*, *S. aureus*, *Salmonella typhimurium*, and *C. perfringens* type C (Wang et al., 2018; Huang X. et al., 2019; Wang et al., 2020). However, the expression of *S100A9* gene was actually controlled by the methylation status of its promoter (Chandra et al., 2018). In our study, *S100A9* gene was down-expressed in transcription and hypermethylated in methylation, and was identified in the Cp infected IR and IS groups, hinting that the hypermethylation *S100A9* gene caused the down expression, which may play some roles in protecting piglets from Cp-induced diarrhea resistance.

Transactivation response element RNA-binding protein *TARBP2* inhibits the catalytic activity of interferon-induced double-stranded RNA (dsRNA)-activated protein kinase (PKR) to regulate stress-induced signaling pathways during viral infections and cell stress, and *TARBP2* is also a regulator of microRNA biogenesis and cellular stress response (Ukhueduan et al., 2021). *TARBP2* has been characterized as a key regulator for promoting or inhibiting cell proliferation and invasion. Research has shown that the *TARBP2* suppresses IFN-beta production and the innate antiviral response, especially in regulating the antiviral signaling pathways of the innate immune system (Ling et al., 2019). *TARBP2* gene, differential expressed and differential DNA methylated, was identified in Cp infected piglets of the IR and IS groups, which may play some important roles in piglet diarrhea resistance.

To further explore the susceptibility and tolerance of piglet resistance to Cp-induced diarrhea, we screened the potential resistant candidates from the IR and IS groups by integrating data of DNA methylation and mRNA expression (Figure 8). There were 14 mRNAs for which methylation of their promoters obviously inversely related to transcriptional repression were identified (Table 4), such as *LCN2*, *TBX21*, and *LBP*. *LCN2* with up-expressed and hypermethylated was identified both in the IR and IS groups after Cp infection. The changes in its promoter methylation and gene expression indicated that *LCN2* may participate in immune response to *C. perfringens* type C stimulation. We also constructed a network of immune-associated mRNAs both different changes in methylation and transcription in the IS vs. IR group (Figure 8), we speculated that these genes may be functionally linked and regulated by promoter methylation level of piglets in response to *C. perfringens* type Cp-induced diarrhea.

*TBX21* is an important transcription factor of adaptive immunity that regulates the Th1/Th2 balance and increasing evidence has pointed to the critical roles in regulating innate immunity, cytokine balance, immune dysregulation, and bacterial infection. A study reported that *pneumococcus* upregulated *TBX21* expression in the respiratory epithelium, knocking down *TBX21* suppressed *pneumococcus*-induced *TLR2* expression (Woo et al., 2014), and a change of *TBX21* may lead to the dysregulation of type I interferon pathways and T cell pathways (Gourh et al., 2009). These studies have proved the adaptive immune regulator *TBX21* participated in regulating host innate immune responses during pathogenic bacterial infections. In this study, differential methylated *TBX21* was identified in piglets in the IS and IR groups after Cp infection, and it was also found that up-regulated and hypermethylated *TBX21* in the IS vs. IR comparative group suggests that hypermethylated *TBX21* gene may cause the down expression in the transcriptional level, while the up-expression of *TBX21* gene may be regulated by other factors, which commonly participate in the process of piglet resistance to Cp-induced diarrhea.

LPS-binding protein (*LBP*) is a plasma protein that transfers LPS to the cell surface CD14 presented on the myeloid lineage, playing the crucial roles in the host innate immune response during the development of inflammatory and infectious-related diseases (Meng et al., 2021). *LBP* is also essential to control bacterial infection. Recently, *LBP* has been shown to potentiate the host immune responses to LPS invade, relieving *Salmonella typhimurium* or *E. coli* induced generation of reactive oxygen species in host macrophages (Sclutt et al., 1999). The LPS-LBP complex can bind to a receptor complex (CD14, MD2, and TLR4) for initiating signal cascade and triggering the secretion of pro-inflammatory cytokines (Gabarin et al., 2021; Won et al., 2021). While accomplishing either blocking *LBP* binding to LPS or binding LPS/LBP complexes to CD14 can protect the host from LPS-induced toxicity (Le Roy et al., 1999). Therefore, in this study, the hyper methylation of *LBP* was significantly negative regulation with its down expression in transcription in susceptible piglets for Cp infection, while the tolerance piglets with high expression may maintain balance of the inflammatory response induced by Cp infection. Considering the functions and expression characteristic of these genes, we believe that *LBP* and *TBX21* are strongly associated with piglet diarrhea induced by Cp infection. We speculated that these genes may be functionally linked and regulated by promoter methylation level of piglets in response to Cp-induced diarrhea, which may play some certain roles in protecting piglet resistance of diarrhea caused by bacterial infection.

In conclusion, we have profiled the landscape of DNA methylation of piglets in response to Cp infection, and analyzed the methylation and transcriptome data to further reveal the potential candidates implicated in the piglet immune response. Our findings have provided insight into the molecular effects of DNA methylation of piglet resistance to *C. perfringens* type C infection, which may contribute to the selection and breeding of piglet diarrhea resistant to *C. perfringens* type C infection.

## Data Availability

The datasets presented in this study can be found in online repositories. The names of the repository/repositories and accession number(s) can be found in the article/Supplementary Material.

## Abbreviations

DMRs, differentially methylated regions; *E. coli*, *Escherichia coli*; GO, gene ontology; KEGG, Kyoto Encyclopedia of Genes and Genomes; mRNA, messenger RNA; RNAseq, RNA sequencing; RRBS, reduced representation bisulfite sequencing.
